# Evaluation of a deep learning-based reconstruction method for denoising and image enhancement of shoulder MRI in patients with shoulder pain

**DOI:** 10.1007/s00330-023-09472-9

**Published:** 2023-02-18

**Authors:** Georg C. Feuerriegel, Kilian Weiss, Sophia Kronthaler, Yannik Leonhardt, Jan Neumann, Markus Wurm, Nicolas S. Lenhart, Marcus R. Makowski, Benedikt J. Schwaiger, Klaus Woertler, Dimitrios C. Karampinos, Alexandra S. Gersing

**Affiliations:** 1 Department of Radiology, Klinikum Rechts Der Isar, School of Medicine, Technical University of Munich, Ismaninger Strasse 22, 81675 Munich, Germany; 2 Philips GmbH Market DACH, Hamburg, Germany; 3 Musculoskeletal Radiology Section, Klinikum Rechts Der Isar, School of Medicine, Technical University of Munich, Munich, Germany; 4 Department of Trauma Surgery, Klinikum Rechts Der Isar, School of Medicine, Technical University of Munich, Munich, Germany; 5 Department of Neuroradiology, Klinikum Rechts Der Isar, School of Medicine, Technical University of Munich, Munich, Germany; 6 Department of Neuroradiology, University Hospital of Munich, LMU Munich, Munich, Germany

**Keywords:** Magnetic resonance imaging, Deep learning algorithm, Compressed SENSE, Shoulder injury

## Abstract

**Objectives:**

To evaluate the diagnostic performance of an automated reconstruction algorithm combining MR imaging acquired using compressed SENSE (CS) with deep learning (DL) in order to reconstruct denoised high-quality images from undersampled MR images in patients with shoulder pain.

**Methods:**

Prospectively, thirty-eight patients (14 women, mean age 40.0 ± 15.2 years) with shoulder pain underwent morphological MRI using a pseudo-random, density-weighted *k*-space scheme with an acceleration factor of 2.5 using CS only. An automated DL-based algorithm (CS DL) was used to create reconstructions of the same *k*-space data as used for CS reconstructions. Images were analyzed by two radiologists and assessed for pathologies, image quality, and visibility of anatomical landmarks using a 4-point Likert scale.

**Results:**

Overall agreement for the detection of pathologies between the CS DL reconstructions and CS images was substantial to almost perfect (*κ* 0.95 (95% confidence interval 0.82–1.00)). Image quality and the visibility of the rotator cuff, articular cartilage, and axillary recess were overall rated significantly higher for CS DL images compared to CS (*p* < 0.03). Contrast-to-noise ratios were significantly higher for cartilage/fluid (CS DL 198 ± 24.3, CS 130 ± 32.2, *p* = 0.02) and ligament/fluid (CS DL 184 ± 17.3, CS 141 ± 23.5, *p* = 0.03) and SNR values were significantly higher for ligaments and muscle of the CS DL reconstructions (*p* < 0.04).

**Conclusion:**

Evaluation of shoulder pathologies was feasible using a DL-based algorithm for MRI reconstruction and denoising. In clinical routine, CS DL may be beneficial in particular for reducing image noise and may be useful for the detection and better discrimination of discrete pathologies.

**Summary statement:**

Assessment of shoulder pathologies was feasible with improved image quality as well as higher SNR using a compressed sensing deep learning–based framework for image reconstructions and denoising.

**Key Points:**

***•*** *Automated deep learning–based reconstructions showed a significant increase in signal-to-noise ratio and contrast-to-noise ratio (p < 0.04) with only a slight increase of reconstruction time of 40 s compared to CS.*

***•*** *All pathologies were accurately detected with no loss of diagnostic information or prolongation of the scan time.*

***•**** Significant improvements of the image quality as well as the visibility of the rotator cuff, articular cartilage, and axillary recess were detected.*

## Introduction

In modern society, shoulder pain is very common and may cause impairment in everyday and work activities [1]. In the general population, acute as well as chronic shoulder pain often originates from rotator cuff pathologies and pathologies of other soft tissue structures [2]. MR imaging is currently the modality of choice for imaging of shoulder pain, due to the high contrast and resolution, in particular if soft tissue pathologies are suspected [3, 4]. MR imaging of the shoulder is often challenging in patients with shoulder pain (breathing artifacts, motion artifacts caused by pain, etc.) and potential artifacts caused by surgery [5, 6]. Furthermore, imaging quality is often corrupted due to increased image noise (e.g., Rician noise) or limited by the surface coil where tissues that lie peripheral to the coil elements are less assessable due to increased noise [7]. As consequence, increased image noise could lead to inaccurate diagnosis or impaired image analysis [7].

Different strategies have previously been introduced in order to reduce image noise including traditional approaches which mainly are based on filtering, transformations or statistical methods, as well as modern DL-based approaches which are based on convolutional neuronal networks (CNNs) and general adversarial networks (GANs) [7–14]. Traditional patch-based denoising methods such as non-local means (NLM) algorithms rely on the self-spatial similarities of natural images and have proven to be compatible with iterative image reconstruction methods based on parallel accelerated imaging, e.g., SENSE and GRAPPA [10, 11, 15–17]. In recent studies, the application of deep learning–based algorithms was suggested for reconstruction of accelerated MR scans as well as for image denoising [12, 14, 18, 19]. Zhang et al created a supervised feed-forward CNN which separates noise from noisy observation and uses residual modules as well as batch normalization to speed up the denoising performance [12]. As a self-learning self-supervised image denoising network, Xu et al proposed the “Noisy-As-Clean” network [13]. This method declares corrupted test images as ground truth (“clean” target) and uses synthetic images, which consist out of small alterations to the corrupted test image in order to train the network. In general, CNNs and GANs apply self-learning reconstruction schemes and have shown promising results in order to reduce image noise and accelerate the MR imaging data acquisition process in contrast to the classic iterative reconstruction schemes [18, 20–24]. These DL-based methods apply reconstruction schemes in order to calculate high-quality images from undersampled MR data.

In this study, we used a reconstruction framework which utilizes a novel CNN to integrate and enhance conventional CS algorithms based on the adaptive-CS-network, previously described by Pezzotti et al [18]. Therefore, the purpose of this study was to assess the diagnostic performance and denoising capabilities of the reconstruction framework which combines PI, CS, and a DL-based algorithm (CS DL) for the assessment of various shoulder pathologies on multiplanar shoulder MRI compared to images reconstructed with CS only.

## Methods and materials

### Study participants

In this prospective study, patients with shoulder pain (*N* = 38, mean age 40 ± 15.2 years, 14 women) that were admitted to the orthopedic and trauma surgery departments between June 2021 and January 2022 were enrolled. The patients presented with shoulder pain due to various pathologies including suspected chronic degenerative changes (*n* = 21), acute trauma (*n* = 9), as well as unclear shoulder pain (*n* = 8). Informed consent was obtained from all study participants prior to inclusion. The study was approved by our institutional review board (Ethics Commission of the Medical Faculty, Technical University of Munich, Germany; ethics proposal number 42/21S). To calculate the appropriate number of study participants, a priori power analysis was performed using data of a preceding study [25]. The data of the SNR calculations between the ankle MRI with CS only and the ankle MRI with the deep learning reconstructed CS was used to simulate a comparison between the two different groups. Finally, a sample size of at least 24 subjects per group was determined to achieve a power  > 0.8. Therefore, we included 38 participants into the study to ensure adequate group sizes for the comparison.

### MR imaging

Each patient underwent a 3-T MRI examination (Ingenia Elition; Philips Healthcare) of the shoulder using a dedicated 16-channel shoulder coil. A clinical routine imaging protocol was used including a triplanar intermediate-weighted (IM) turbo spin-echo (TSE) sequence with spectral fat saturation, a sagittal T2-weighted TSE sequence, and a coronal T1-weighted TSE sequence. Detailed scan parameters are displayed in Table 1. All acquired data were reconstructed with standard CS and CS DL.

**Table 1:** Sequence parameters of the sequences used in this study

Sequence	Coronal IM/SPIR	Axial IM/SPIR	Sagittal IM/SPIR	Sagittal T2	Coronal T1
Echo time (ms)	50	50	50	80	19
Repetition time (ms)	2400	2450	2400	2500	730
Acceleration factor	2.5	2.5	2.5	2.5	2.5
TSE factor	16	16	16	16	5
Field of view (mm^3^)	160 × 160 × 83	160 × 160 × 83	160 × 160 × 83	160 × 160 × 108	160 × 160 × 76
Voxel size (acquisition, mm^3^)	0.4 × 0.54 × 3.0	0.4 × 0.54 × 3.0	0.4 × 0.54 × 3.0	0.35 × 0.49 × 3.0	0.35 × 0.45 × 3.0
Voxel size (reconstructed, mm^3^)	0.28 × 0.28 × 3.0	0.28 × 0.28 × 3.0	0.28 × 0.28 × 3.0	0.24 × 0.24 × 3.0	0.24 × 0.24 × 3.0
Slice thickness (mm)	3	3	3	3	3
Slice number	26	31	28	30	23
Acquisition time (min)	3.39	3.24	3.18	3.11	2.3

#### C-SENSE DL

The CS DL reconstructions investigated in this study are based on an adaptive-CS-network, as previously described by Pezzotti et al [18]. This network utilizes a novel CNN to integrate and enhance conventional CS algorithms. The adaptive-CS-network is an advancement of the deep learning–based iterative shrinkage-thresholding algorithm (ISTA) network proposed by Zhang and Ghanem [26]. It integrates multiscale sparsification in a problem-specific learnable manner. Further, the CNN-based sparsification approach is combined with the image reconstruction approach of CS and therefore, ensures data consistency. Prior information such as coil sensitivity distribution and location of the image background are automatically incorporated as well. The adaptive-CS-network therefore combines parallel imaging, compressed sensing, and deep learning into a single algorithm and replaces the wavelet transform by a CNN as sparsifying transform in the CS algorithm. The adaptive-CS-network used in this study was initially trained with a dataset of approximately 740,000 MR images from various anatomical regions acquired using 1.5-T and 3-T MR imaging. The algorithm was refined to run on standard reconstruction hardware, in contrast to the previously reported network [18]. Reconstruction was performed on the scanner and the reconstructions took approx. 80 s for the CS DL reconstructions compared to approximately 40 s for the standard CS reconstructions.

### Quantitative image analysis

**Fig. 1:**
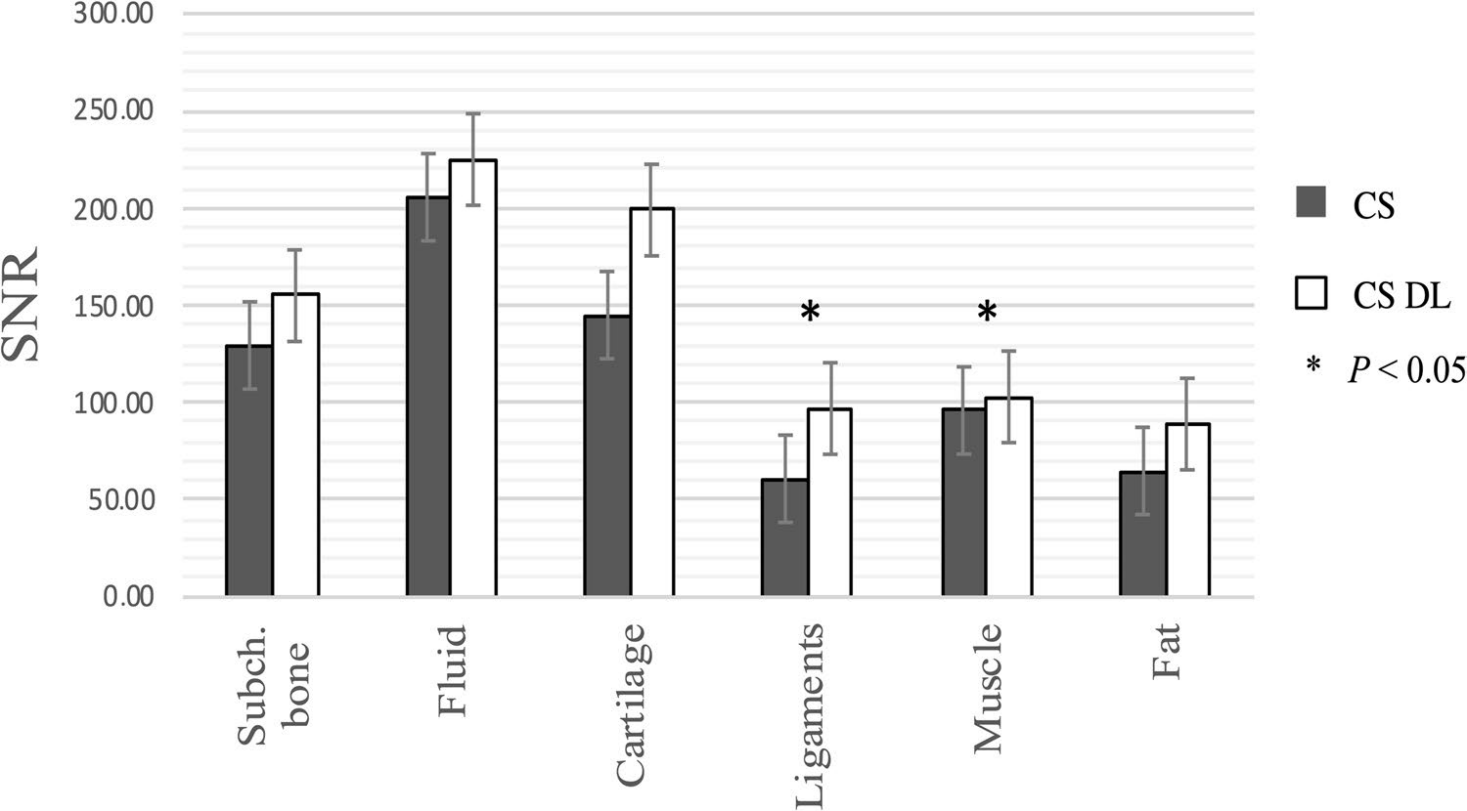
Calculated SNR for subchondral bone, fluid, cartilage, ligaments, muscle, and fat. Significant higher SNR values were seen for ligaments and muscle of the CS DL reconstructions (ligaments *p* = 0.01, muscle *p* = 0.04)

**Fig. 2:**
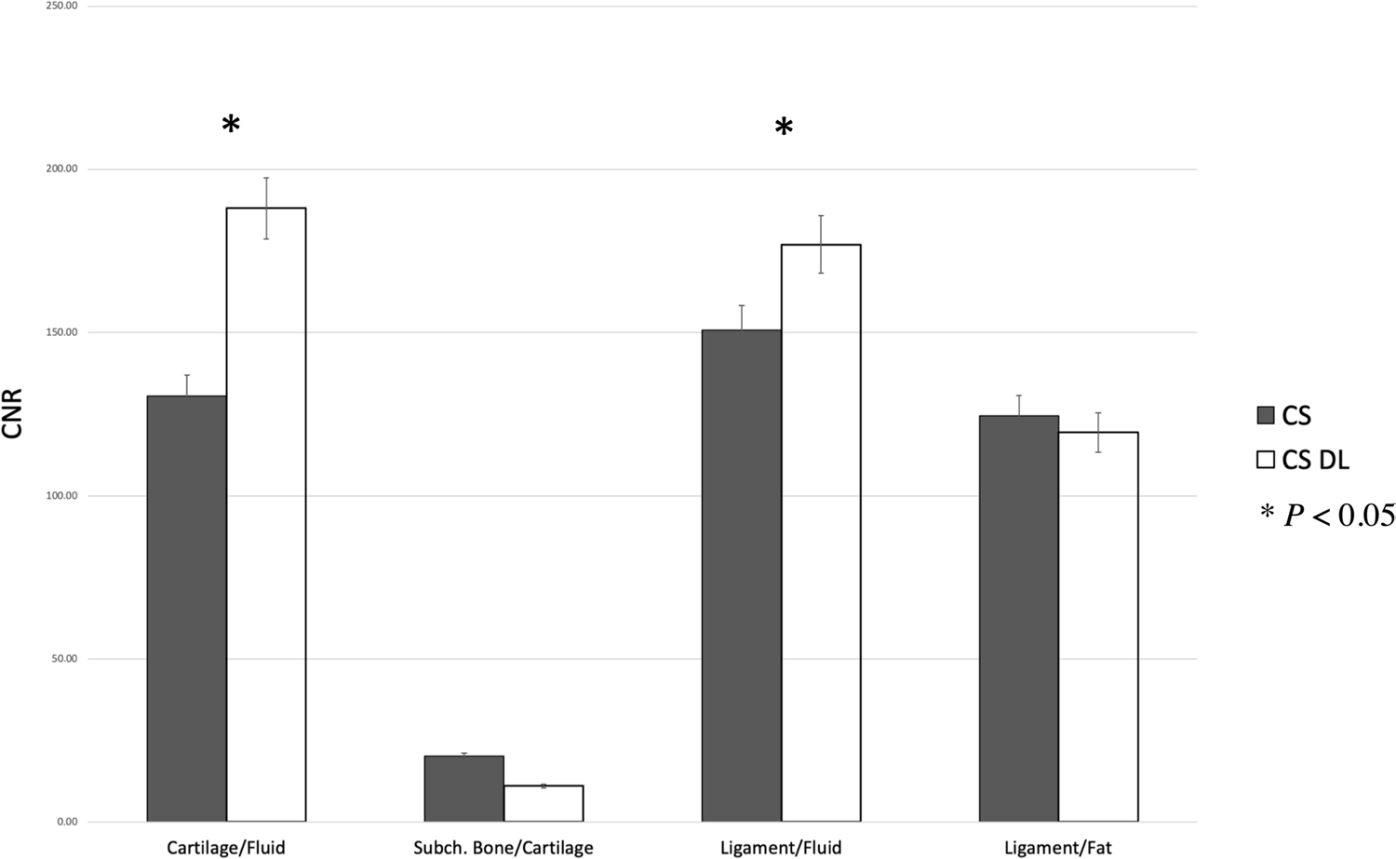
Calculated CNR for cartilage/fluid, subchondral bone/cartilage, ligament/fluid, and ligament/fat. CNR values of cartilage/fluid and ligament/fluid of the CS DL reconstructions were significantly higher compared to standard CS images (*p* < 0.05)

Signal-to-noise (SNR) and contrast-to-noise (CNR) values were calculated for CS only and CS DL using an established subtraction method (Figs. 1 and 2) [27–29]. Therefore, sequences of ten patients were acquired twice in the same exam session. The repeated sequences were subtracted using the inbuild MRI software to create the noise maps in which regions of interest were placed in the same location on three consecutive slices. SNR was calculated as previously described [27]:

$${SNR}_{Diff}=\frac{1}{\sqrt{2}}\times \frac{\left({SI}_{1}+{SI}_{2}\right)}{{SI}_{3}\times \sqrt{\frac{\pi }{2}}},$$

*SI*_1_ measures the signal intensity of the ROI in the first series, *SI*_2_ the signal from the ROI in the second series, and SI3 the ROI from the ROI in the noise maps. Following SNR calculation was then performed for muscle, ligaments, joint fluid, subchondral bone, and fat. CNR was calculated by subtracting the SNR of tissue 1 with the SNR of tissue 2 and was calculated for cartilage/fluid, subchondral bone/cartilage, ligament/fluid, and ligament/fat.

### Semi-quantitative image analysis

Image readings were performed by two experienced radiologists separately and independently and blinded to all clinical information (Y.L. with 4 years of experience in musculoskeletal imaging and J.N. with 10 years of experience in musculoskeletal imaging). Readings were performed on a PACS work station certified for clinical use (IDS7 21.2, Sectra). The standard CS images and CS DL reconstructed images were read with at least 3 weeks in between readings, respectively. For intra-reader reproducibility, 10 patients were assessed once again after 4 weeks by both radiologists.

The CS and CS DL images were analyzed for the visibility of anatomical landmarks graded with a 4-point Likert scale (1 = inadequate, 2 = adequate, 3 = good, 4 = excellent) based on the extent of the partial volume effect, blurring, image noise, signal inhomogeneity, and discrimination from adjacent structures [27]. Following landmarks were assessed for visibility: rotator cuff tendons and muscles, biceps anchor, long biceps tendon, rotator cuff interval, AC joint, articular cartilage, axillary recess, and labrum. Overall image quality was assessed also using a 4-point Likert scale based on the overall image expression. Furthermore, the rotator cuff tendons, long biceps tendon, rotator cuff muscles, glenoid and humeral cartilage, bone, bursa, AC joint, glenoid labrum, and joint capsule were assessed for visibility and presence of pathologies. The bone was assessed for bone marrow edema, subchondral cysts, or osseous defects such as Bankart and Hill-Sachs lesions [30]. Rotator cuff tendons were assessed for tendinopathy including subacromial impingement, rotator cuff tendinitis/tendinosis, and calcific tendonitis, and the fatty infiltration was graded according to Goutallier et al [31]. The biceps tendon was assessed for tendinopathic changes, dislocation, as well as lesions of the biceps pulley. Anatomical variations of the labrum were graded according to Kanatli et al [32] (for detailed information about the gradings of abnormalities, see Table 2). The overall diagnostic confidence of the readings was also graded with a 4-point Likert scale based on how confident the readers were regarding the evaluation of pathologies. Severeness of motion artifacts, blurring, or image noise was semi-quantitatively classified into no, little, and severe artifacts.

**Table 2:** Assessed abnormalities/pathologies of the shoulder and detected numbers

Parameters	Grading and frequency (*n*, %)
Bone	Normal signal: 24 (63%)
	Bone marrow edema: 1 (3%)
	Subchondral cysts: 3 (8%)
	Tuberculum majus fracture: 2 (5%)
	Hill-Sachs defect: 1 (3%)
	Bankart defect: 7 (18%)
Rotator cuff tendons	No pathology: 10 (26%)
	Tendinopathy: 23 (60%) Partial tear: 4 (11%)
	Complete tear: 1 (3%)
Rotator cuff muscle	No pathology: 29 (76%)
	Fatty infiltration: 0 (0%)
	Acute injury: 9 (24%)
Biceps	No pathology: 22 (58%)
	Tendinopathy: 15 (39%)
	Dislocation: 0 (0%)
	Tear: 0 (0%)
	SLAP: 0 (0%)
	Pulley: 1 (3%)
Cartilage	Normal signal: 31 (82%)
	Abnormal: 7 (18%)
Bursa	Normal signal: 28 (74%)
	Inflamed: 10 (26%)
AC joint	Normal joint: 10 (26%)
	Osteoarthritis: 28 (74%)
Effusion	No effusion: 31 (82%)
	Joint effusion: 7 (18%)
Labrum	Normal labrum: 30 (79%)
	Anatomical normal variant: 1 (3%)
	Lesion: 7 (18%)

### Statistical analysis

The data were analyzed using IBM SPSS, version 25.0 (IBM Corp.). All statistical tests were performed two sided, and a level of significance (*α*) of 0.05 was used for all tests. A Shapiro–Wilk test was performed to test for normal or non-normal distribution of the data. A paired *t*-test was used for comparison of normally distributed numerical variables and a Wilcoxon signed-rank test was used to compare non-normally distributed numerical and categorical variables between CS and CS DL image assessments. McNemar’s test was used to assess for binary categorical variables. In order to assess the inter- and intra-observer agreement, Cohen’s kappa was used for ordinal scaled data and inter-class correlation coefficient using a two-way random-effects model with absolute agreement for nominal scaled data. The values can be interpreted as poor (0), slight (0.0–0.2), fair (0.21–0.40), moderate (0.41–0.60), substantial (0.61–0.80), and almost perfect (0.81–1.00) [33, 34]. For all measurements, 95% confidence intervals (CI) were calculated.

## Results

### Assessment of shoulder pathologies/abnormalities

**Fig. 3:**
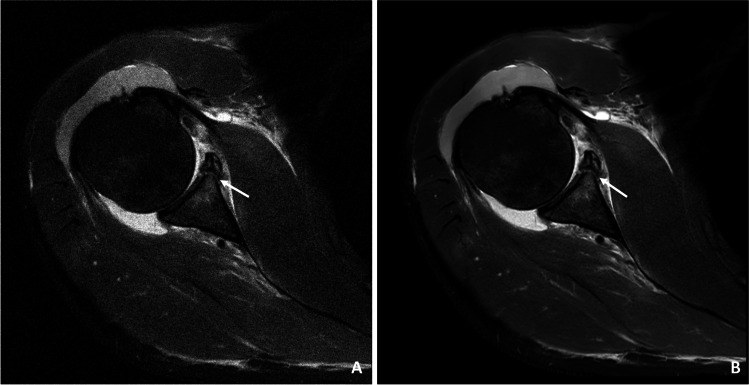
**A** Transversal IM-weighted TSE sequence of a 34-year-old participant with an acute Bankart fracture. **B** High-resolution CS DL reconstruction of the transversal image with markedly reduced image noise and a clear discrimination of the glenoid fracture borders (white arrows)

**Fig. 4:**
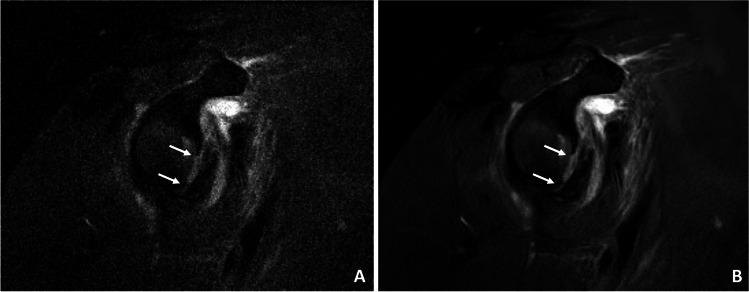
A 46-year-old patient with anterior fracture dislocation of the right shoulder. **A** Standard sagittal IM-weighted sequence with TSE showing increased noise in the whole images. **B** CS DL reconstruction of the IM-weighted TSE sequence with markedly reduced overall noise and smoother borders of the osseous Bankart fragment (white arrows)

**Fig. 5:**
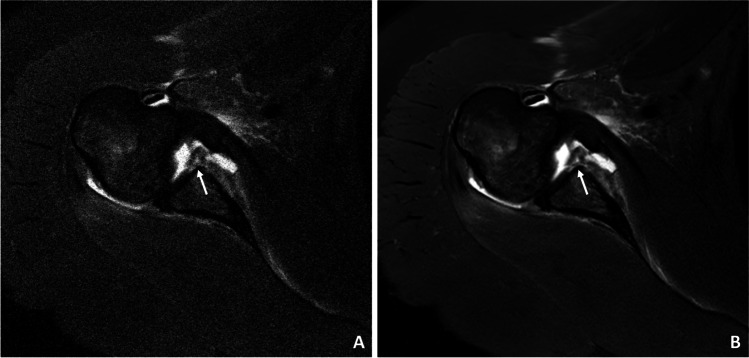
A 64-year-old patient after acute shoulder dislocation with decentered humeral head and lesion of the anterior inferior labrum. (**A**) Note the reduced noise and smooth display of the labral defect (white arrows) in the high-resolution CS DL reconstructions (**B**)

**Fig. 6:**
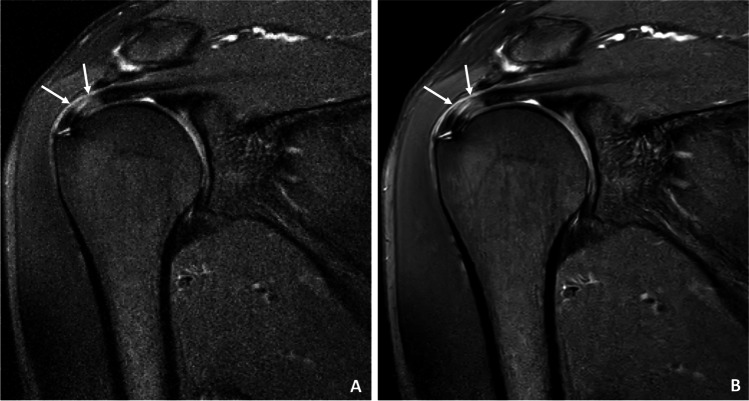
A 54-year-old patient with tendinopathic changes of the rotator cuff, in particular the supraspinatus tendon. **A** Standard coronal T1-weighted sequence with TSE. **B** CS DL reconstruction of the T1-weighted TSE sequence with overall reduced noise and smoother discrimination of the tendinopathy of the supraspinatus tendon (white arrows)

Almost all pathologies were accurately detected on the CS DL reconstructions by both readers compared to the standard CS images with no significant difference (readers 1 and 2: *κ* 0.95 (95% confidence interval 0.82–1.00)). In total, 9 acute fractures were detected, of which 7 were osseous Bankart lesions (Figs. 3, 4, and 5) and two were humerus fractures. Moreover, four partial tears (Fig. 6) and one complete tear of one or more rotator cuff tendons were detected. Joint effusion was detected in 7 patients and 28 patients showed signs of an osteoarthritis of the AC joint. Detailed numbers and the distributions of pathologies are listed in Table 2. The overall diagnostic confidence of pathologies detected on the CS DL images was higher compared to CS in both readers, and this finding reached the statistical level of significance for one reader (reader 1 CS: 3.2 ± 0.1, CS DL 3.8 ± 0.2, *p* = 0.18; reader 2 CS: 2.9 ± 0.2, CS DL 3.7 ± 0.1, *p* = 0.04, Table 3). Compared to the standard CS images, the overall image quality of the CS DL reconstructions was rated significantly higher (reader 1 CS 2.8 ± 0.3, CS DL 3.7 ± 0.1, *p* = 0.02; reader 2 CS 2.7 ± 0.1, CS DL 3.8 ± 0.3, *p* = 0.01). No significant differences were detected for the ratings of motion artifacts (reader 1 CS: 3.7 ± 0.4, CS DL 3.8 ± 0.4, *p* = 0.23; reader 2 CS: 3.7 ± 0.4 and CS DL 3.8 ± 0.5, *p* = 0.25). There were no severe motion artifacts detected in the CS DL reconstructions as well as in the CS only images.

**Table 3:** Comparison of the mean image quality, motion artifacts, and diagnostic confidence

	Reader 1			Reader 2		
	CS	CS DL	*p* value	CS	CS DL	*p* value
Diagnostic confidence	3.2 ± 0.1	3.8 ± 0.2	0.18	2.9 ± 0.2	3.7 ± 0.1	0.04
Motion artifacts	3.7 ± 0.4*	3.8 ± 0.4*	0.23	3.7 ± 0.4*	3.8 ± 0.5*	0.25
Overall image quality	2.8 ± 0.3	3.7 ± 0.1	0.02	2.7 ± 0.1	3.8 ± 0.3	0.01

Data are presented as means ± standard deviations

4-point Likert scale (4 = excellent; 1 = inadequate)

^*^Motion artifacts graded with a 3-point scale: 2 = severe, 3 = little, 4 = none

### Visibility of anatomical features

The overall visibility of anatomical regions recorded was higher for the CS DL reconstruction by both readers, yet no statistical significance was reached (reader 1 CS DL 2.5 ± 0.2, 3.1 ± 0.3, *p* = 0.17; reader 2 CS DL: 2.4 ± 0.4 and CS: 3.2 ± 0.2, *p* = 0.31). A significant increase of the visibility was seen for the rotator cuff tendons (reader 1 CS 2.3 ± 0.4, CS DL 3.5 ± 0.3, *p* = 0.04; reader 2 CS 2.2 ± 0.4, CS DL 3.5 ± 0.3 *p* = 0.03), articular cartilage (reader 1 CS: 2.3 ± 0.4, CS DL 3.5 ± 0.2, *p* = 0.03; reader 2 CS: 2.2 ± 0.2, CS DL 3.5 ± 0.3, *p* = 0.02), rotator interval (reader 1 CS: 2.4 ± 0.4, CS DL 3.5 ± 0.1, *p* = 0.02; reader 2 CS: 2.4 ± 0.1 CS DL 3.4 ± 0.2, *p* = 0.03), and axillary recess (reader 1 CS: 2.0 ± 0.6, CS DL 3.2 ± 0.4, *p* = 0.02; reader 2 CS: 2.1 ± 0.4, CS DL 3.2 ± 0.3, *p* = 0.04). An increase of visibility was also detected for the biceps anchor (reader 1 CS: 2.1 ± 0.3, CS DL 3.1 ± 0.4, *p* = 0.06; reader 2 CS: 2.3 ± 0.3, CS DL 3.2 ± 0.2, *p* = 0.05), AC joint (reader 1 CS: 2.6 ± 0.3, CS DL 3.6 ± 0.2, *p* = 0.26; reader 2 CS: 2.7 ± 0.3, CS DL 3.6 ± 0.2, *p* = 0.34), and labrum (reader 1 CS: 2.6 ± 0.3, CS DL 3.6 ± 0.2, *p* = 0.26; reader 2 CS: 2.7 ± 0.3, CS DL 3.6 ± 0.2, *p* = 0.34), yet these results did not reach the level of significance (Table 4). Only a slight increase was seen in the visibility of the long biceps tendon, muscle, and bone. None of the anatomical regions was rated lower in the CS DL reconstructions than in the standard CS images.

**Table 4:** Visibility of anatomical regions of the shoulder

	Reader 1			Reader 2		
Anatomical regions	CS	CS DL	*p* value	CS	CS DL	*p* value
Rotator cuff tendons	2.3 ± 0.4	3.5 ± 0.3	0.04	2.2 ± 0.4	3.5 ± 0.3	0.03
Long biceps tendon	2.8 ± 0.5	3.5 ± 0.4	0.59	2.8 ± 0.4	3.4 ± 0.4	0.53
Biceps anchor	2.1 ± 0.3	3.1 ± 0.4	0.06	2.3 ± 0.3	3.2 ± 0.2	0.05
Rotator interval	2.4 ± 0.4	3.5 ± 0.1	0.02	2.4 ± 0.1	3.4 ± 0.2	0.03
AC joint	2.6 ± 0.3	3.6 ± 0.2	0.26	2.7 ± 0.3	3.6 ± 0.2	0.34
Articular cartilage	2.3 ± 0.4	3.5 ± 0.2	0.03	2.2 ± 0.2	3.5 ± 0.3	0.02
Axillary recess	2.0 ± 0.6	3.2 ± 0.4	0.02	2.1 ± 0.4	3.2 ± 0.3	0.04
Labrum	2.2 ± 0.3	2.9 ± 0.2	0.21	2.2 ± 0.3	2.9 ± 0.2	0.34
Bone	2.5 ± 0.3	3.2 ± 0.5	0.69	2.3 ± 0.4	3.1 ± 0.4	0.74
Muscle	2.5 ± 0.4	3.3 ± 0.6	0.55	2.6 ± 0.5	3.3 ± 0.4	0.62

Data are presented as means ± standard deviations

4-point Likert scale (4 = excellent; 1 = inadequate)

### Quantitative image analysis

Significant higher SNR values were detected for ligaments and muscle of the CS DL reconstructions compared to the standard CS images (ligaments *p* = 0.01, muscle *p* = 0.04). Although the SNR values of the CS DL reconstructions were generally higher, no statistical significance was reached for fat, joint fluid, and subchondral bone (Fig. 1). Comparing the CNR values, the CNR values of cartilage/fluid and ligament/fluid of the CS DL reconstructions were significantly higher compared to standard CS images (cartilage/fluid *p* = 0.02 and ligament/fluid *p* = 0.03, respectively; Fig. 2).

### Inter- and intra-reader agreement

Inter-reader agreement for the detection and grading of pathologies was substantial to almost perfect (*κ* 0.89 (95% confidence interval 0.71–1.00)). Agreement for the grading of the visibility of anatomical regions was also substantial to almost perfect (*κ* 0.94 (95% confidence interval 0.89–1.00)). For intra-reader reliability, both readers reassessed the images of 10 patients after at least 4 weeks. The intra-reader agreement was overall substantial to almost perfect (range *κ* 0.84 to 1.00) for both readers. All acute pathologies were once more accurately identified on the CS DL reconstructions (*κ* 1.00 (95% CI 1.00–1.00) for both readers). Only in two patients the tendinopathy of the supraspinatus tendons was rated as partial tears instead (*κ* 0.84 (95% confidence interval 0.75–1.00)).

## Discussion

In this study, the application of a compressed sensing deep learning–based framework for image reconstruction and denoising was investigated and shown to be feasible and to improve image quality while remaining accurate regarding the assessment of various pathologies of the shoulder. Images with CS DL reconstructions overall showed a significantly higher image quality and a higher diagnostic confidence indicating that the better image quality and visibility of anatomical landmarks may be useful for the visualization and differentiation of pathologies. The increased image quality and denoising in the CS DL reconstructions may be particularly beneficial in patients with subtle findings and increased image noise. Almost all pathologies were identically detected on the CS DL reconstructions as in the standard CS images, with no loss of information due to the DL-based reconstruction algorithm. Furthermore, the visibility of the articular cartilage, AC joint, and rotator cuff tendons was rated higher in the CS DL images compared to standard CS reconstructions. The reduced image noise also improved the discrimination between tissues which may improve the detection and assessment of e.g. acute fractures or degenerative changes. Due to the denoising, significant higher SNR values were detected for ligaments and muscle of the CS DL reconstructions which further increases the image quality. CNR for cartilage/fluid and ligament/fluid was significantly higher in the CS DL reconstruction. In general, the reduced image noise of the CS DL reconstruction may be particularly useful when assessing more peripheral pathologies, e.g., injury to the biceps tendon, muscle, or scapula, and might help identify discrete pathologies which otherwise would be difficult to discriminate.

In recent studies, different approaches for deep learning–based enhancement of reconstruction quality and noise reduction in CS imaging had been proposed. Manimala et al successfully implemented a CNN for fast denoising of sparse MR images corrupted with Rician noise [19]. The algorithm exploits patch-based processing in order to update and refine the dictionary of weights. Furthermore, it can be employed without estimating the noise level and preserves the local structures better compared to traditional methods like NLM [19]. Chaudhari et al implemented a deep learning–based 3D CNN called “DeepResolve” to reconstruct small-slice high-resolution images from acquired thicker slices and was able to achieve superior quantitative and qualitative diagnostic performance [35]. Quan et al developed a convolutional autoencoder and GAN which employ deeper generator and discriminator networks with cyclic data consistency loss for interpolation of the under-sampled *k*-space data [36]. This enables faster image acquisition but also enhanced reconstruction quality using a chained network. In a retrospective study, Koch et al successfully utilized a neuronal network for denoising of shoulder and hip MRI which was trained with a supervised learning approach using pairs of high-spatial-resolution high-signal-to-noise ratio images and synthesized low-resolution low-signal-to-noise ratio images [37].

To our knowledge, none of the abovementioned studies evaluated shoulder pathologies and anatomical structures with traumatic and non-traumatic pathologies prospectively in a larger patient cohort. Furthermore, with the CS DL algorithm used in this study, image reconstruction was performed automatically during acquisition with no further time-consuming postprocessing needed. We found that CS DL reconstructions are generally applicable and reliable, yet, depending on the pathology and patient compliance, it has a larger or smaller benefit on image quality. Furthermore, our study was conducted in a clinical routine setup and our framework is applicable on every standard 3 T MRI scanner and can be implied on scanning protocols without the need of special hardware.

In our current work, the CS DL reconstructions were not accelerated, meaning there was no difference in scan times. Both CS and CS DL images were reconstructed from the same data, to minimize differences and confounders due to quality variations in the acquired data for both techniques. However, in future work, a thorough optimization of CS DL sequences is warranted to investigate the impact of CS DL on further image acceleration or increased resolution compared to what currently is possible with standard CS.

There are certain limitations to our study which need to be addressed. First, we examined an inhomogeneous patient collective of 38 patients with various disorders and injuries of the shoulder. Studies with larger study cohorts with multiple cases of one pathology are needed in the future in order to show the applicability of the CS DL reconstructions for various pathologies. For comparison, the standard CS images were used as standard of reference but no further modality, e.g., arthroscopy, was available. Therefore, confirmation of diagnosis was not verified by an external standard of reference. Only in the acute trauma cases with occurring fractures (*n* = 9) an additional conventional CT scan was available.

The assessment of shoulder pathologies was feasible, and the image quality and the SNR were significantly improved, while remaining accurate regarding the assessment of the pathologies when using a compressed sensing deep learning–based framework for image reconstructions. The reduced image noise improved the quality as well as visibility of anatomical landmarks compared to the standard CS reconstructions and might help with the detection of even discrete pathologies. In clinical routine, this automated reconstruction and denoising technique might be particularly useful when applied to challenging MRI acquisitions, e.g., in traumatic shoulder injuries.

## Abbreviations

CNR: Contrast-to-noise ratio
CS: Compressed SENSE
CS AI: Compressed SENSE artificial intelligence
CNN: Convolutional neural network
ETL: Echo train length
GAN: Generative adversarial network
IM: Intermediate-weighted
PI: Parallel imaging
SNR: Signal-to-noise-ratio
TSE: Turbo spin-echo

## Declarations

### Guarantor

The scientific guarantor of this publication is PD. Dr. med. Alexandra S. Gersing.

### Statistics and biometry

No complex statistical methods were necessary for this paper.

### Methodology

• prospective

• case–control study

• performed at one institution
